# High-flow nasal cannula oxygenation reduces desaturation risk during diagnostic flexible bronchoscopy under deep sedation: a randomized controlled trial

**DOI:** 10.3389/fmed.2025.1729660

**Published:** 2026-01-08

**Authors:** Ming Wang, Longfei Wang, Xuefei Zhou, Wanquan Ming, Cheng Sheng, Rong Xu, Youhua Wu, Yongbin Chen, Yonghua Zhang, Yunfei Cao

**Affiliations:** 1 Health Science Center, Ningbo University, Ningbo, China; 2 Department of Anesthesiology, Beilun People’s Hospital of Ningbo, Ningbo, China; 3 Department of Anesthesiology, Qilu Hospital (Qingdao), Cheeloo College of Medicine, Shandong University, Qingdao, China; 4 Department of Pulmonary, Beilun People’s Hospital of Ningbo, Ningbo, China

**Keywords:** deep sedation, desaturation, diagnostic flexible bronchoscopy, end-tidal carbon dioxide, high-flow nasal cannula oxygenation

## Abstract

**Background:**

Deep sedation for flexible bronchoscopy (FB) improves procedural conditions but exacerbates desaturation risks. High-flow nasal cannula (HFNC) may mitigate this situation, yet efficacy under deep sedation remains unproven.

**Methods:**

In this randomized trial (ChiCTR2400083597), 340 ASA I-II patients undergoing FB under deep sedation (MOAA/S ≤ 1) received conventional nasal cannula (5 L/min; NC) or HFNC (25/45/65 L/min). The primary outcomes were the incidence of intraoperative desaturation (SpO₂ < 90% > 10s) and intraoperative nadir SpO₂ value. The intraoperative VAS scores for cough that reflect the stimulation inhibition as a result of deep sedation were used as secondary outcome. Other evaluated outcomes included the EtCO₂ values before induction and after awakening, incidence of intraoperative hypertension/hypotension, postoperative adverse events, as well as the willingness to undergo re-examination.

**Results:**

All procedures were completed in approximately 5 min, without requiring the application of laryngeal mask airway or endotracheal intubation. HFNC groups showed significantly lower desaturation incidence (HF25:16.87%, HF45:12.05%, HF65:5.00%) versus NC (57.14%, *p* < 0.000), with weak flow-dependence. All HFNC cohorts maintained higher nadir SpO₂ (*p* < 0.000) and reduced CO₂ retention versus NC (*p* < 0.05). Cough scores were uniformly low (median VAS:1–1.5), with >97% willingness for repeat FB.

**Conclusion:**

The application of HFNC can markedly lower the risks of desaturation during FB under deep sedation in a weak flow-dependent manner, while partially mitigating carbon dioxide accumulation, enabling safer deep sedation without intubation.

## 1 Introduction

Unlike gastrointestinal endoscopy, flexible bronchoscopy (FB) involves direct tracheobronchial manipulation, which induces intense airway irritation. Patients may experience severe discomforts, including violent coughing, asphyxia, and a sense of impending death, as well as critical adverse events such as desaturation, circulatory overload, and malignant arrhythmias (1, 2). So sedation is recommended currently by most guidelines or expert consensus to be offered to all patients undergoing FB without contraindications, aiming to mitigate stress responses, optimize procedural conditions, and improve patient comfort (3–5).

Although mild-to-moderate sedation is generally recommended during FB to preserve spontaneous breathing, clinical evidence indicates desaturation incidence still approaches 56% even at this depth (6). Desaturation constitutes an inherent risk of FB due to the airway-sharing requirement of the bronchoscope and sedation-induced respiratory depression, with potential life-threatening severity. Consequently, the currently advocated mild-to-moderate sedation protocol represents a compromise between anesthetic efficacy and patient safety. This strategic limitation of sedation prioritizes preserving adequate spontaneous respiration and the patient’s responsiveness, thus enabling rapid corrective intervention during oxygen desaturation events via temporary withdrawal of the flexible bronchoscope and/or verbal commands to arouse the patient (7).

However, the mild-to-moderate sedation regimen frequently fails to effectively suppress the intense airway reflexes and severe coughing provoked by FB, exposing patients to recurrent procedural stimuli. This approach not only increases technical difficulty for the FB operator but also adversely impacts tolerance of patients, ultimately diminishing compliance with subsequent endoscopic examinations. While increasing sedation depth may theoretically enhance patient comfort and procedural conditions, this approach carries significant risks of exacerbated respiratory depression and elevated desaturation incidence (8, 9). Therefore, performing FB under deep sedation with conventional oxygen supplementation has been consistently considered unsafe and is not recommended in prevailing clinical guidelines and expert consensus documents.

The emergence of novel oxygen delivery systems, particularly high-flow nasal cannula (HFNC) oxygenation, presents a potential paradigm shift in this clinical situation. HFNC delivers heated, humidified, and oxygen-enriched air via nasal cannula at flow rates up to 70 L/min, offering multiple physiological benefits including maintenance of constant FiO_2_, apneic oxygenation, pharyngeal dead space reduction, decreased work of breathing, positive end-expiratory pressure (PEEP) generation, and enhanced mucociliary clearance and gas exchange (10, 11). These physiological advantages, combined with the system’s simplicity and patient comfort, establish HFNC as an optimal solution not only for respiratory failure patients but also for perioperative oxygenation and ventilation support (11–13).

Accumulating clinical evidence demonstrates the effectiveness of HFNC in FB, with multiple randomized controlled trials reporting significant reductions in desaturation incidence and enhanced nadir SpO_2_ values during procedures (14). Nevertheless, all these investigations were confined to mild-to-moderate sedation levels, with no existing literature addressing applications under deep sedation. Considering that deep sedation typically induces severe respiratory depression, current FB guidelines advocate for introducing airway devices (such as laryngeal masks or endotracheal intubation) with assisted ventilation. While HFNC, which has no effective ventilation function, is not yet mentioned. For this reason, in this randomized controlled trial, we aim to evaluate whether HFNC is beneficial for FB under deep sedation, with a focus on providing adequate oxygenation with acceptable carbon dioxide accumulation.

## 2 Materials and methods

### 2.1 Study subjects

This single-center, prospective, randomized controlled trial was conducted at a 1,100-bed tertiary teaching hospital following approval by the Ethics Committee of Beilun People’s Hospital (Approval No. 2024-17-K). The study was registered with the Chinese Clinical Trial Registry (ChiCTR2400083597). All participants provided written informed consent in compliance with the Declaration of Helsinki.

From July 2024 to May 2025, 427 adult patients (aged 18–60 years; ASA class I–II) undergoing diagnostic FB—including bronchial biopsy, brushing, and bronchoalveolar lavage—were screened, with 340 meeting eligibility criteria. The primary indications for FB were pneumonia (76.4%), bronchiectasis (10.3%), pulmonary shadow (6.7%), hemoptysis (4.5%), and others (2.1%). Exclusion criteria comprised: (1) Patients with severe sleep apnea syndrome (apnea–hypopnea index > 40) or baseline desaturation with measured peripheral capillary oxygen saturation (SpO_2_) < 90% in room air, (2) patients with a history of alcohol abuse or current use of any psychiatric medication, (3) patients who had neurologic disorders or other conditions contributing to difficulty in assessing a conscious response, (4) pregnant and lactating women, (5) patients refusing to give informed consent.

### 2.2 Procedure and sedation

All patients scheduled for diagnostic FB were fasted for 6 h for solids and for 2 h for clear fluids preoperatively. Prior to the procedure, they received 15-min nebulization with 5 mL of 2% lidocaine and 2 mL of terbutaline sulfate solution. In the endoscopy room, continuous monitoring included electrocardiography, noninvasive blood pressure, respiratory rate, and pulse oximetry. End-tidal carbon dioxide (EtCO₂) was measured pre-sedation using a mainstream capnograph (EMMA™, Radius PCG; PN 3639, Sweden). The oxygen delivery mode for each group was as follows: the control group received 5 L/min oxygen via unheated nasal cannula, while three HFNC groups received 100% oxygen at 36 °C through an OptiFlow™ THRIVE system (Fisher & Paykel Healthcare, New Zealand) at flows of 25 L/min (HF25), 45 L/min (HF45), and 65 L/min (HF65) throughout the procedure. After 2 min of preoxygenation, sedation was initiated and all four groups received identical deep sedation protocols: 0.05 mg/kg midazolam (Nhwa Pharmaceutical Co., Ltd., Jiangsu, China, lot number: H19990027) was dispensed with normal saline to 10 mL and bolus administered by slow intravenous injection within 20 s. 1-min later, a bolus of 23 μg/kg alfentanil (Yichang Renfu Pharmaceuticals Co., Ltd., Yichang, China, lot number: H20190034) was dispensed with normal saline to 30 mL and injected intravenously and completed in 60 s. If the target depth of sedation [Modified Observer’s Assessment of Alertness/Sedation (MOAA/s) score ≦ 1] was not achieved, propofol (Fresenius Kabi Austria GmbH Co., Ltd., Austria, lot number: 16TF1204) would be titrated as a rescue. No additional sedatives were administered during the procedure. Then, FB procedure was performed by two experienced bronchoscopists (a chief physician and an associate-chief physician in the department of respiratory at our hospital) using EVIS LUCERA BF-260 series bronchoscope (BF260, BF1T260 and BF-P260F; Olympus; Tokyo, Japan) via the nasal route, and 2% lidocaine was sprayed through the bronchoscope channel to enhance topical anesthesia with the “spray-as-you-go” technique at the glottis, main bronchus, and carina (4 mL each site), respectively. Various diagnostic procedures, including bronchial biopsy, bronchial brushing, and bronchoalveolar lavage, were performed based on individual clinical conditions.

During FB, supplemental propofol (20 mg boluses) was administered by the anesthesiologist if inadequate sedo-analgesia was observed. Propofol can be repeated as needed. Intraoperative hypertension (systolic blood pressure > 160 mmHg) or hypotension (<90 mmHg) was treated with intravenous urapidil or dopamine 1 mg per time until hemodynamic stability was achieved. For oxygen desaturation (SpO₂ < 95%), a jaw thrust maneuver was initiated. Desaturation (SpO₂ < 90% for >10 s) triggered temporary mask ventilation; persistent cases required advanced airway management (laryngeal mask insertion or tracheal intubation) at the anesthesiologist’s determination, who could manage the patient’s airway in any way they deemed to be appropriate in the best interest of the patients.

Procedure completion was confirmed by bronchoscopists upon endoscope removal. Immediate reversal was achieved with naloxone (3 μg/kg) and flumazenil (5 μg/kg). After confirming full consciousness recovery, EtCO₂ was measured immediately, and when the stability of vital signs was confirmed, patients were transferred to the recovery room, and continuously monitored and received supplemental oxygen (5 L/min) through a conventional nasal catheter for 15 min, then transferred to inpatient ward with a 3-h constant monitoring.

### 2.3 Data collection and measured outcomes

Perioperative data were collected, including the EtCO_2_ values before induction and after awakening, intraoperative changes in vital signs, cough severity evaluated by visual analog scale (VAS) with a score from 0 to 10 indicating no cough to intolerable cough resulting in procedural interference, and postoperative follow-up data.

The primary outcomes were the incidence of intraoperative desaturation (defined as peripheral saturation of oxygen [SpO_2_] < 90% lasting more than 10 s), as well as intraoperative nadir SpO_2_ value. The intraoperative VAS scores for cough that reflect the stimulation inhibition as a result of deep sedation were used as secondary outcome. Other evaluated outcomes included the EtCO₂ values before and after the operation, incidence of intraoperative hypertension/hypotension, postoperative adverse events, as well as the willingness to undergo re-examination.

### 2.4 Randomization, sample size estimation and statistical methods

The randomization was performed by random number table from the SPSS 23.0, and 340 enrolled patients were attached with sequential inclusion numbers and randomized into four groups containing 85 patients each (*n* = 85). A single-blind design was employed due to the different oxygen supply modes and oxygen flow rates of the four groups. Anesthesiologist, bronchoscopists and nurses involved in the procedure of flexible bronchoscopy, were not blinded to the study, whereas patients were blinded as well as researchers responsible for outcome assessment and statistical analysis.

For sample size calculation, a pilot study was conducted to determine the sample size and PASS 15.0 software (version 15.0; NCSS, LLC, Kaysville, UT, United States) was used. The preliminary data showed that the incidences of desaturation during FB under deep sedation in the four groups were 60, 20, 5, and 5%, respectively (*n* = 20). The results indicated that a sample size of only 13 subjects per group would provide 90% power with an alpha error of 0.05 to detect a linear trend among groups in the proportion of subjects with desaturation using a two-sided *Z*-test with continuity correction. However, a sample size of 76 subjects per group was needed to distinguish the differences among the three HFNC groups, and to compensate for possible dropouts (10%), the sample size was increased to 85 subjects per group.

Categorical variables are presented as counts and proportions, analyzed using chi-square tests or Fisher’s exact tests as appropriate. Continuous variables were tested for normality via the Kolmogorov–Smirnov method and visual histogram inspection. Normally distributed data are reported as mean ± standard deviation (SD) and analyzed with one-way ANOVA test. Multiple comparisons among the four groups were computed based on LSD test, and the comparison of EtCO_2_ values before and after FB was made using paired Student’s *t*-tests. Non-normally distributed data are expressed as median and interquartile range (IQR), analyzed with the Kruskal-Wallis H test. All analyses were performed using IBM SPSS Statistics 25.0 (Windows) without imputation for missing data, and statistical significance was defined as *p* < 0.05.

## 3 Results

### 3.1 Study population and baseline characteristics

**Figure 1:**
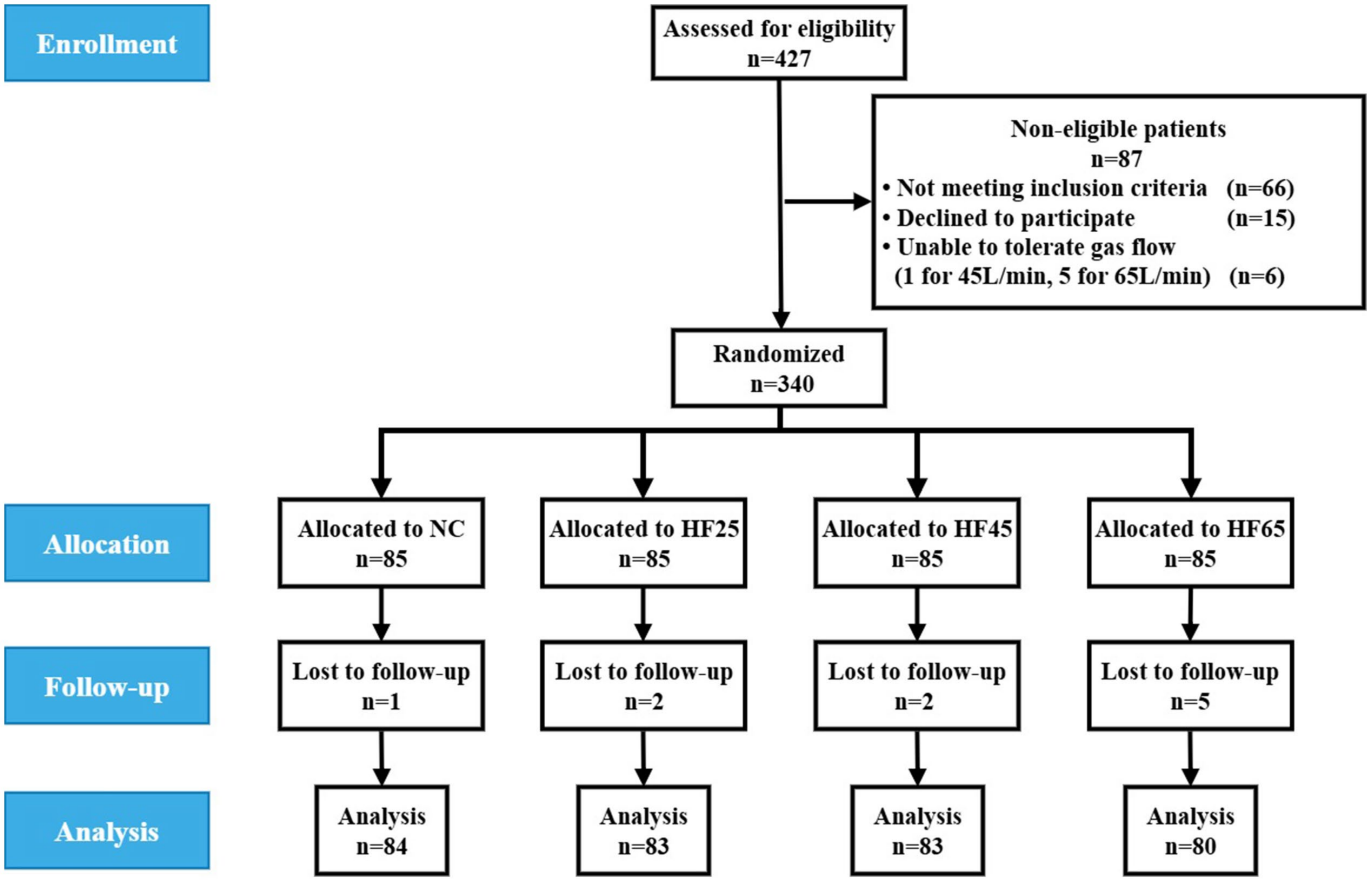
Flow diagram of the study.

Out of screened 427 patients, 340 patients were enrolled in this study and distributed randomly into four groups, as shown in Figure 1. During the procedure of diagnostic FB, a total of 10 patients withdrew from the trial. Among them, in the control group, 1 patient was converted to mass resection due to endobronchial masses found unexpectedly during FB procedure. In the HFNC groups, 2 patients in the HFNC25 group withdrew from the trial, one was converted to treatment due to an endobronchial mass, and the other converted to ultrasound-guided transbronchial needle biopsy due to intrabronchial abnormalities. In the HFNC45 group, 2 patients withdrew from the trial, one was converted to transoral FB procedure due to bilateral nasal meatus stenosis, the other one was converted to pulmonary embolism treatment due to massive hemorrhage after biopsy. Five patients in the HFNC65 group withdrew from the trial. One was converted to transoral FB procedure due to bilateral nasal meatus stenosis, one patient was converted to mass resection due to endobronchial masses, two patients converted to ultrasound-guided transbronchial needle biopsy due to intrabronchial abnormalities, and one was lost in postoperative follow-up as shown in Figure 1.

In terms of demographic characteristics, there were no significant differences among the four groups with respect to gender, age, weight, body mass index, and ASA physical status (*p* > 0.05), as shown in Table 1. The primary indication for FB was pneumonia, followed by bronchiectasis, pulmonary shadow and hemoptysis. Diagnostic procedures included bronchoalveolar lavage, bronchial brushing and bronchial biopsy. The indications and procedures were uniformly distributed in four groups, and no significant differences were observed among the groups (*p* < 0.05), as shown in Table 1.

**Table 1:** Patient characteristics, indications and brochoscopic procedures.

Groups	Group NC *n* = 84	Group HF25 *n* = 83	Group HF45 *n* = 83	Group HF65 *n* = 80	*p* value
Sex (M/F)	51/33	51/32	41/42	50/30	0.281
Weight (kg)	63.80 ± 12.20	64.66 ± 12.85	65.63 ± 11.66	64.54 ± 12.90	0.823
Height (cm)	165.27 ± 8.54	166.60 ± 6.93	165.47 ± 6.77	165.60 ± 8.28	0.685
Age (years)	43.08 ± 11.17	42.73 ± 11.66	46.13 ± 11.91	45.18 ± 12.35	0.188
BMI (kg/m^2^)	23.24 ± 3.39	23.17 ± 3.57	23.92 ± 3.73	23.41 ± 3.62	0.529
ASA (I/II) (n)	53/31	45/38	47/36	42/38	0.535
Indications for FB					
Pneumonia	68 (80.5%)	62 (74.70%)	60 (72.29%)	62 (77.50%)	0.587
Bronchiectasis	11 (13.10%)	9 (10.84%)	12 (14.46%)	2 (2.50%)	0.056
Lung shadow	1 (1.19%)	6 (7.23%)	8 (9.64%)	7 (8.75%)	0.120
Hemoptysis	3 (3.57%)	3 (3.61%)	2 (2.41%)	7 (8.75%)	0.277
Miscellaneous	1 (1.19%)	3 (3.61%)	1 (1.20%)	2 (2.50%)	0.652
Procedures for FB (n)					
BAL	4	4	4	9	0.310
BAL + BBr	74	69	71	65	0.645
BAL + BBr + BBi	6	10	8	6	0.673

Data are presented as mean ± SD, or number of patients (%). ASA, American Society of Anesthesiologists; BMI, body mass index; FB, Flexible bronchoscopy; BAL, Bronchoalveolar lavage; BBr, Bronchial brushing; BBi, Bronchial biopsy.

### 3.2 Intraoperative outcomes

**Figure 2:**
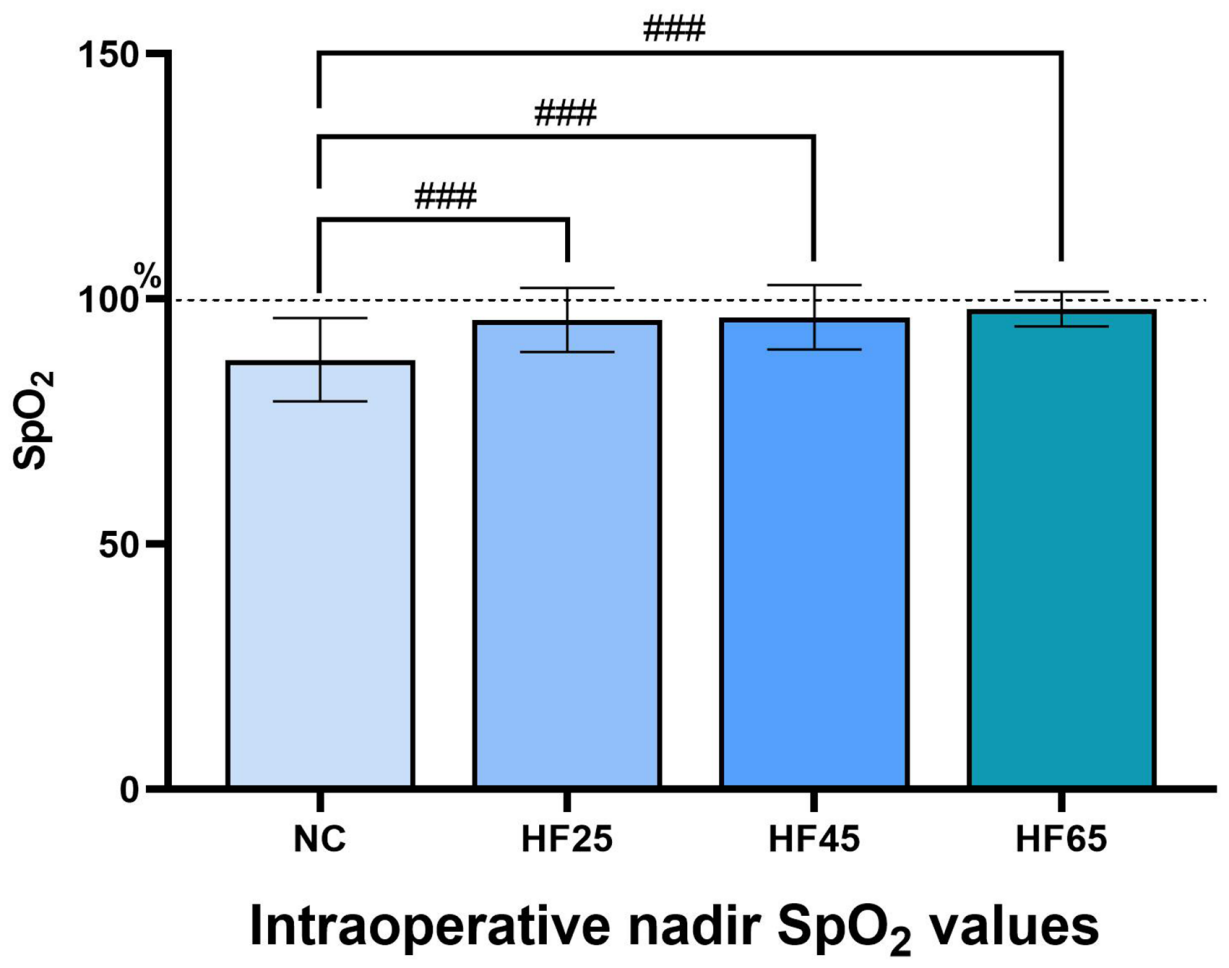
Intraoperative nadir SpO_2_ values in four groups. Compared with NC group, ns: *p* > 0.05, #: *p* < 0.05, ##: *p* < 0.01, ###: *p* < 0.001.

The comparison of the incidence of desaturation as the primary outcome was shown in Table 2. There was no difference in baseline SpO_2_ before pre-oxygenation among the four groups. However, the incidence of intraoperative desaturation (SpO_2_ < 90%) in the NC group (57.14%) was significantly higher than those in the three HFNC groups (*p* < 0.000). Among them, the incidence of desaturation under deep sedation in the three HFNC groups was all less than 20%. For intra-group comparison of HFNC groups, the incidence of desaturation in the HF65 group (5.00%) was significantly lower than that in the HF25 group (16.87%), with statistical significance (*p* = 0.016). However, there were no significant differences between the HF65 group and the HF45 group, or between the HF45 group and the HF25 group. These results suggested that HFNC can significantly reduce the incidence of desaturation and had a weak flow-dependence manner. As for the comparison of intraoperative nadir SpO_2_ value, all three HFNC groups were significantly higher than the control group (*p* < 0.000) in Figure 2. But there were no statistically difference among the three HFNC groups. Although patients in the control group (NC group) required more remedial interventions for desaturation, no severe adverse event was observed throughout the study, and none required laryngeal mask insertion or endotracheal intubation.

**Table 2:** The clinical outcomes and drugs dosage during FB procedure.

Groups	Group NC *n* = 84	Group HF25 *n* = 83	Group HF45 *n* = 83	Group HF65 *n* = 80	*P* value
Baseline SpO_2_ values	97.50 ± 1.85	97.37 ± 1.92	97.30 ± 2.20	96.95 ± 2.10	0.346
FB operating time (min)	5.48 ± 2.73	5.67 ± 2.44	5.04 ± 2.63	5.71 ± 2.86	0.343
Primary outcomes					
Incidences of intraoperative desaturation (SpO_2_ < 90%)	48 (57.14%)^***^	14 (16.87%)^###^	10 (12.05%)^###△^	4 (5.00%)^###*^	0.000
Intraoperative nadir SpO_2_ values	86 (80, 95.75)^***^	99 (95, 100)^###^	99 (96, 100)^###△^	99 (98, 100)^###△^	0.000
Secondary outcomes					
Cough VAS score	1.5 (0.0, 3.0)	1.0 (0.0, 2.0)	1.0 (0.0, 2.0)	1.0 (0.0, 2.0)	0.541
Other outcomes					
EtCO_2_ before sedation	33.01 ± 5.57	32.95 ± 5.56	34.12 ± 6.07	34.33 ± 5.24	0.256
EtCO_2_ after awakening	39.79 ± 4.89^*^	37.84 ± 5.75^#^	37.96 ± 6.26^#△^	37.75 ± 5.30^#△^	0.057
Intraoperative hypertension	2 (2.38%)	1 (1.20%)	3 (3.61%)	1 (1.25)	0.793
Intraoperative hypotension	8 (9.52%)	8 (9.64%)	8 (9.64%)	9 (11.25%)	0.980
Midazolam (mg)	3.19 ± 0.60	3.25 ± 0.65	3.28 ± 0.58	3.24 ± 0.65	0.784
Alfentanil (μg)	1446.19 ± 280.67	1482.53 ± 291.07	1494.28 ± 270.26	1477.50 ± 296.38	0.728
Propofol (mg)	42.14 ± 34.19	48.31 ± 40.78	49.04 ± 42.47	39.00 ± 36.44	0.276

Data are presented as mean ± SD, or number of patients (%), or median (interquartile range). SpO2, hemoglobin oxygen saturation; EtCO2, end-tidal carbon dioxide; VAS, visual analog scale.

LSD tests: compared with NC group, ^□^*p* > 0.05, ^#^*p* < 0.05, ^##^*p* < 0.01, ^###^*p* < 0.001; compared with HF25 group, ^△^*p* > 0.05, ^*^p < 0.05, ^**^p < 0.01, ^***^p < 0.001.

The comparison of cough VAS scores served as the secondary outcome. The intraoperative VAS scores for cough were low across all four groups. Among the 330 subjects included in the final statistical analysis, only 8 patients (2.4%) had a score >3, with only one patient reaching a score of 5. In contrast, 98 patients (29.7%) had a score of 0, indicating no cough, while the remaining patients experienced mild cough with scores ranging from 1 to 3. These findings indicate that effectively inhibited the intense airway stress caused by FB manipulation, and there was no instance of suspension or abandonment of the bronchoscopy procedure due to severe cough.

EtCO_2_ values, as one of the supplementary outcomes, intra-group comparisons showed that the EtCO_2_ values were significantly higher at the end of surgery (after awakening) in all groups as compared to preoperative baseline values, suggesting that there was CO_2_ accumulation after deep sedation in all four groups. Intergroup comparisons showed that there was no difference in the preoperative EtCO_2_ baseline values among the four groups, but significant differences were observed in the EtCO_2_ values at the end of surgery, the EtCO_2_ values at the end of the surgery in all the three HFNC groups were significantly lower than that in the control group, but there was no significant difference among the three HFNC sub-groups. These results indicated that HFNC could alleviate CO_2_ accumulation to a certain extent, but with no flow-dependent manner.

The incidence of intraoperative hypertension is very rare, while in contrast, intraoperative hypotension is more common, demonstrating the inhibitory effect of deep sedation on circulation. However, there were no statistically differences among the four groups in terms of the VAS score of cough, the incidence of intraoperative circulation fluctuations (intraoperative hypertension or hypotension), the FB operating time and the dosage of sedative drugs, as shown in Table 2.

### 3.3 Postoperative follow-up and adverse events

The follow-up at 6 h after surgery showed that, all the patients in the four groups had no postoperative recall (the unintended experience and memory of procedural events), and the willingness to return for a second FB examination under the same sedo-analgesia was more than 97%, indicating that the deep sedation regime adopted in this study had a high degree of comfort and acceptability for patients. Some patients may experience transient dizziness or fatigue after the surgery, with an incidence rate ranging from 15.66 to 31.25%. However, no special treatment was required, and most of the symptoms disappear spontaneously within 2 h after surgery, Postoperative nausea and vomiting were rare, and also need not special treatment. Moreover, the inter-group statistical analysis showed that there were no statistically differences among the four groups in terms of postoperative recall, unwillingness to return, and postoperative discomforts including lethargy, dizziness, nausea or vomiting (*p* > 0.05, Table 3).

**Table 3:** Postoperative follow-up and adverse events.

Groups	Group NC *n* = 84	Group HF25 *n* = 83	Group HF45 *n* = 83	Group HF65 *n* = 80	*P* value
Postoperative discomforts					
Nausea	1 (1.19%)	1 (1.20%)	2 (2.41%)	3 (3.75%)	0.584
Vomiting	0 (0%)	1 (1.20%)	0 (0%)	2 (2.50%)	0.149
Fatigue	17 (20.24%)	17 (20.48%)	25 (30.12%)	22 (27.50%)	0.341
Dizziness	17 (20.24%)	15 (18.07%)	13 (15.66%)	25 (31.25%)	0.075
Postoperative follow-up					
Postoperative recall	0 (0%)	0 (0%)	0 (0%)	0 (0%)	/
Unwillingness to return	2 (2.38%)	1 (1.20%)	2 (2.41%)	1 (1.25%)	0.889

## 4 Discussion

While HFNC has been extensively studied in FB procedures under mild-to-moderate sedation, no randomized controlled trials to date have systematically evaluated its potential benefits in the context of deep sedation. This might be the first study to indicate that HFNC generates significant improvements in both oxygenation and ventilation parameters during deep sedation FB, mainly manifested as an attenuation of desaturation risk with concurrent alleviation of carbon dioxide (CO₂) retention. Critically, HFNC enables safe implementation of non-intubated deep sedation bronchoscopy, optimizing procedural conditions and providing effective respiratory support even during anticipated severe sedation-induced respiratory depression.

Undoubtedly, deep sedation more effectively suppresses intense airway irritation and severe cough responses during FB, providing optimal operating conditions and enhanced patient comfort. However, severe respiratory depression and unacceptably high desaturation risk associated with deep sedation significantly outweigh these benefits (15, 16). Our results also confirm that during bronchoscopy under deep sedation, the cough VAS score was very low (median: 1–1.5), and the cough response was significantly lower than that reported in our previous study with a mild-to-moderate sedation regime for bronchoscopy, as well as less intraoperative hypertension and tachycardia (17). However, the desaturation incidence reached 57.14% in controls receiving conventional nasal catheter oxygen supply (5 L/min). This aligns with Wei et al.’s reported 84% desaturation incidence during deeply sedated (MOAA/S 1–2) diagnostic FB, where higher rates may relate to the inclusion of ASA grade 3 cases and propofol-based regimens (9). Due to the concerns of oxygenation, most guidelines recommend mild-to-moderate sedation for FB operation, as deep sedation with conventional oxygen supply measures is generally unsafe without endotracheal intubation or LMA assistance—but airway devices that increase complexity and cost (10, 18).

HFNC, as an advanced oxygen delivery system, enables non-intubated bronchoscopy under deep sedation while maintaining spontaneous respiration, which has been confirmed in our previous study with elderly patients undergoing FB examination (19). In this study, we find that HFNC significantly decreases desaturation risk during FB under deep sedation, with incidence rates showing a declining trend as flow rates increased from 25 to 65 L/min. This is very similar to previously reported outcomes of HFNC-supported FB under mild-to-moderate sedation (20–22). Specifically, Zhang et al. observed flow-dependent desaturation reduction during propofol-sulfentanil moderate sedation (flow rate from 10 to 60 L/min) (22). In patients receiving FB under deep sedation, however, our investigation revealed attenuated HFNC flow dependence for FB desaturation prevention: minimal intraoperative SpO₂ showed no significant differences among three subgroups, while desaturation incidence differed solely between HF25 and HF65 cohorts. This may be due to the fact that most patients unconsciously open their mouths during our FB procedure, especially after receiving sedation. Existing evidence confirms HFNC reduces FB-related desaturation through FiO₂ elevation and positive airway pressure generation (20, 23), subsequent studies reveal that though mouth opening has little effect on FiO_2_ (24), HFNC-generated airway pressure can be significantly diminished with open-mouth breathing (mean: 2.3 cm H₂O at 45 L/min when closed vs. near-zero when open) (25). Since HFNC’s clinical benefits -including improved oxygenation, ventilation-perfusion matching, and reduced respiratory work -largely depend on airway pressure maintenance (26), open-mouth states during FB may attenuate both HFNC’s anti-desaturation efficacy and flow-dependent therapeutic mechanisms.

The efficacy of HFNC in significantly reducing the desaturation risk of FB under deep sedation, despite lacking active ventilation capacity, is quite beyond our initial expectations. This effect may mainly derive from two functions of HFNC, namely oxygen reservoir expansion via pre-oxygenation and apnoeic oxygenation (24–27). Apnea is prevalent during FB sedation, a 100% apnea occurrence was recorded by Minami et al. under fentanyl-midazolam sedation during bronchoscopy (26). HFNC’s oxygen reservoir expansion and apnoeic gas exchange are critical for bridging sedation-induced temporary respiratory depression, thereby preventing both desaturation events and ventilation intervention requirements. Clinical evidence validates its capacity to enhance oxygenation and extend safe apnea duration during anesthesia induction (11). Admittedly, other benefits of HFNC, such as nasopharyngeal dead space reduction, positive end-expiratory pressure (PEEP) effect generation, end-expiratory lung volume augmentation, and alveolar ventilation preservation, also contribute to improving oxygenation (11, 16, 20). Just as Patel A et al. elucidated, HFNC synergizes apnoeic oxygenation with continuous positive airway pressure and gaseous exchange through flow-dependent dead space flushing, achieving remarkable oxygenation improvement and desaturation risk reduction (28). However, even at maximal flow rates (65 L/min), HFNC still demonstrates a desaturation incidence of approximately 5% during deep sedation FB. This confirms that HFNC cannot entirely eliminate desaturation risks or replace assisted ventilation. Intensive intraoperative monitoring and preparation for assisted/controlled ventilation are still required.

CO₂ accumulation during deep sedation constitutes a significant clinical concern beyond desaturation risk (26, 28). Sedative agents can induce varying degrees of respiratory depression and hypoventilation, with established evidence demonstrating frequent PaCO₂ elevation during bronchoscopy, particularly under deep sedation rather than mild or moderate sedation (3, 4, 17). And the application of HFNC, which lacks effective ventilation capacity, raises ongoing debate regarding its role in modulating (even exacerbating) CO₂ retention during FB (29, 30). However, to date, the impact of HFNC on CO₂ accumulation during deep sedation of FB has not reported in the literature. The results of this study indicated significant end-procedure EtCO₂ elevation in all groups, confirming that respiratory depression and hypoventilation also exist even after awakening from deep sedation, and the application of HFNC could alleviate the accumulation of CO₂ to a certain extent, which was independent of the flow rate (25–65 L/min). These observations align partially with prior mild-to-moderate sedation studies suggesting HFNC’s dual benefit in oxygenation improvement and CO₂ reduction (31–33). Irmak et al. indicates HFNC minimizes CO_2_ retention primarily through enhanced CO_2_ clearance. This functions by continuously displacing exhaled upper airway air with low- CO_2_ fresh gas, reducing rebreathing (32). A controlled trial confirmed HFNC’s superiority over standard nasal prongs in facilitating CO_2_ removal and lowering Pt CO_2_ (33). Additionally, HFNC alleviates respiratory depression and CO2 retention by reducing respiratory work and lung tissue metabolic demand while improving oxygenation and respiratory muscle function (31, 34). However, contrasting findings have been reported regarding HFNC’s effect on EtCO₂. While Irfan et al. (14) and Douglas et al. (15) found no significant difference in EtCO₂ levels between HFNC and conventional oxygen therapy post-surgery, patients with CO₂ retention tendencies may develop inadequate ventilation when receiving oxygen supplementation, particularly with high-flow 100% oxygen, resulting in elevated EtCO₂ levels. Su et al. reviewed five randomized controlled trials involving 257 patients and drew conclusions that HFNC may reduce the incidence of hypoxemic events and improve oxygenation in patients undergoing FB but has no significant effect on the EtCO_2_ value at the end of the surgery (34). Divergent findings regarding HFNC’s influence on CO₂ retention during FB sedation may reflect methodological differences in sedation administration and airway patency (particularly the open-mouth state during FB procedures). Notably, in the open-airway model, HFNC at 10 L/min achieves optimal CO₂ clearance—but this effect diminished in closed system thus requiring higher flow rates for equivalent efficacy (35). Meanwhile, the open-mouth state compromises HFNC’s ability to generate flow-dependent pressure in the upper airways, thereby limiting its efficacy in promoting ventilation and reducing CO₂ retention (36, 37). This may explain the observed absence of flow-dependence in CO₂ alleviation in this study, though these speculations remain unsubstantiated and require further mechanistic investigation.

This study presents several limitations. Firstly, although our data analysis indicates a significant advantage of HFNC in reducing the risk of desaturation during FB examination under deep sedation, larger sample sizes, multicenter studies, as well as well-designed randomized controlled clinical trials are needed to confirm and support these findings. Secondly, the results of this study are only applicable to the relatively healthy population with ASA grade 1 or 2. Although patients with obvious obesity were not excluded in this study, the actually enrolled patients had a BMI up to 34 kg/m_2_, so the results of this study are not applicable to patients with obvious obesity. Thirdly, the cocktail regimen of deep sedation used in this study consists of a basal hypnotic dose of midazolam, a relative higher dosage (more than 20ug /kg) of alfentanil, and a remedial dose of propofol, our previous studies and other literature showed that its anti-stress effect was more significant, while the respiratory inhibitory effect was relatively mild (38, 39). However, it is quite different from the conventional sedation regimen recommended by guidelines or expert consensus. Therefore, whether the results are also applicable to other sedation regimens needs to be verified by further research. Finally, arterial blood gas was not analyzed in this study. Although EtCO_2_ values were monitored at two time points before and after surgery, and the existence of respiratory depression and CO_2_ accumulation were observed in this study, it did not fully reflect the CO_2_ changes throughout the whole FB procedure.

## 5 Conclusion

The major risks of deep sedation with preserved spontaneous breathing during FB should be emphasized as respiratory depression, desaturation and CO₂ accumulation. In this study, HFNC improved oxygenation and ventilation by significantly reducing desaturation risk in a weakly flow-dependent manner and partially alleviating CO_2_ retention, thus enhancing sedation efficacy and patient comfort. However, HFNC cannot completely eliminate desaturation risk or replace assisted ventilation, necessitating continuous monitoring and preparation for advanced ventilatory support.

## Data Availability

The original contributions presented in the study are included in the article/supplementary material, further inquiries can be directed to the corresponding author.
